# Characterization of Feces-Derived Bacterial Membrane Vesicles and the Impact of Their Origin on the Inflammatory Response

**DOI:** 10.3389/fcimb.2021.667987

**Published:** 2021-05-07

**Authors:** Nader Kameli, Reitske Borman, Carmen Lpez-Iglesias, Paul Savelkoul, Frank R.M. Stassen

**Affiliations:** ^1^ School of Nutrition and Translational Research in Metabolism (NUTRIM), Department of Medical Microbiology, Maastricht University Medical Center, Maastricht, Netherlands; ^2^ Department of Medical Microbiology, Faculty of Applied Medical Sciences, Jazan University, Jazan, Saudi Arabia; ^3^ Microscopy CORE Lab, The Maastricht Multimodal Molecular Imaging Institute M4I, Faculty of Health, Medicine, and Life Sciences, Maastricht University, Maastricht, Netherlands; ^4^ Dept of Medical Microbiology and Infection Control, Amsterdam University Medical Centers, location VUmc, Amsterdam, Netherlands

**Keywords:** intestinal microbes, membrane vesicles, bead-based flow cytometer, inflammatory response, bacterial phyla

## Abstract

The human gastrointestinal tract harbors a diverse and complex microbiome, which interacts in a variety of ways with the host. There is compelling evidence that gut microbial dysbiosis, defined as an alteration of diversity and abundance in intestinal microbes, is an etiological factor in inflammatory bowel disease (IBD). Membrane vesicles (MVs), which are nano-sized particles released by bacteria, have been found to interact with the host and modulate the development and function of the immune system. As a result MVs have been suggested to play a critical role in both health and disease. In this study we developed a method to isolate, characterize and assess the immunoreactivity of heterogeneous populations of MVs from fecal samples (fMVs) of healthy volunteers. We successfully isolated 2*10^9^-2*10^10^ particles/ml from 0.5 gram of feces by using a combination of ultrafiltration and size exclusion chromatography (SEC) from 10 fecal samples. Bead-based flowcytometry in combination with tunable resistive pulse sensing (TRPS) provided a reliable method for (semi-)quantitative determination of fMVs originating from both Gram-positive and Gram-negative bacteria, while transmission electron microscopy confirmed the presence of fMVs. Real time 16s PCR on bacterial cell fractions or isolated fMVs DNA of the most common phyla (*Firmicutes, Bacteroidetes, Actinobacteria and Proteobacteria*) revealed differences in the relative abundance between bacteria and the fMVs. Moreover, fMVs evoke the release of TNF- by THP-1 cells in a dose-dependent matter. Also, a significant positive correlation was found between Actinobacteria/-Proteobacteria derived vesicles and the release of TNF-. It has become increasingly clear that fMVs could provide an additional layer to the definition of homeostasis or dysbiosis of the microbiota. The current study supports their potential involvement in the intestinal homeostasis or inflammatory disorders and provides putative interesting incentives for future research.

## Introduction

The human gastrointestinal tract harbors a diverse and complex microbiome, which interacts in a variety of ways with the host. Recent advances in metagenomics and high throughput screening have demonstrated a correlation between the microbiome composition and both health and disease (Round and Mazmanian, 2009; Li etal., 2017; Thomas etal., 2017). There is compelling evidence that gut microbial dysbiosis, defined as an alteration in the diversity and abundance of intestinal microbes, is an etiological factor in IBD. Moreover, evidence is accumulating suggesting additional associations between microbial dysbiosis and a variety of diseases affecting the pulmonary and cardiovascular systems (Dang and Marsland, 2019; Tang etal., 2019) as well as the liver (Minemura and Shimizu, 2015) and even the brain (Foster and McVey Neufeld, 2013). Molecular mechanisms underlying these effects have been elucidated to some extent. Besides pathogenic effects the hosts microbiota also has positive effects on the health of the host by inducing colonization resistance, e.g. by out-competing pathogenic bacteria for space and trophic resources, or by regulation of mucosal immunity. Also, the expansion and differentiation of regulatory T-cells by microbiota-derived short-chain fatty acids seems crucial in maintaining immune homeostasis (Smith etal., 2013; Tanoue etal., 2016). Detrimental effects in disease progression may result from the entry of gut bacteria or their metabolites into the circulation because of an impaired intestinal mucosal barrier. More recently it has been suggested that membrane vesicles, naturally released by most types of bacteria, may contribute to both health and disease and as such be important mediators by which the microbiome affects the host (Cecil etal., 2019; Macia etal., 2019).

The release of nano-sized membrane vesicles (MVs) by bacteria has been addressed in different conditions including bacterial pathogenesis, host interaction and vaccination production. MVs are typically 20 to 300 nm nanostructures naturally released by both Gram-negative and Gram-positive bacteria during different phases of growth (Brown etal., 2015; Schwechheimer and Kuehn, 2015). Depending on their origin and the context for release, these membrane vesicles govern functions in survival, communication and defense (Schwechheimer and Kuehn, 2015; Liu etal., 2018; Volgers etal., 2018). Interaction between bacterial MVs and the host modulates the development and function of the immune system, which has been suggested to play a critical role in both health and disease. This is supported by recent studies which investigated the immunoregulatory role of MVs and showed pro- as well as anti-inflammatory effects of these MVs (Shen etal., 2012; Hickey etal., 2015; Chu etal., 2016; Durant etal., 2020). Interestingly, studies have indicated that differential inflammatory responses were observed, when cells were exposed to a combination of MVs from different bacterial taxa (Kang etal., 2013). This suggests that the immunoregulatory implications of the gut bacterial MVs are dependent on their specific location in the gut and that the complex underlying interactions rather than independent players might explain their involvement in homeostasis and diseases. However, besides a few examples (Choi etal., 2015; Park etal., 2018) extensive knowledge on how MVs derived from the intestinal microbiota affect host physiology or disease this is still lacking. Therefore, a detailed analysis of these vesicles is highly recommended. In this study we developed a method to isolate, characterize and assess the immunoreactivity of heterogeneous populations of MVs from fecal samples of healthy volunteers.

## Material and Methods

### Bacterial Culture and Fecal Samples

The bacterial strains used were *E. coli* ATCC 25276 and *S. aureus* ATCC 29213. All strains were cultured at 5% CO_2_ and 37C overnight on blood agar plates. Ten fecal samples from healthy volunteers were obtained and processed at the Medical Microbiology department (Maastricht University Medical Center, The Netherlands). The fecal samples were collected by the healthy volunteers at home and brought to the hospital within 24hours after defecation. The samples were aliquoted into 0.5g each and frozen directly at 80C for further analyses (samples were processed within three weeks).

### Vesicles Isolation From Bacterial Cultures

Vesicles were isolated from late log-phase to early stationary phase after 15h of culturing by using a combination of ultrafiltration and size exclusion chromatography (SEC) as previously described (Kolling and Matthews, 1999; Boing etal., 2014; Volgers etal., 2017). Briefly, 35ml of tryptone soya broth (TSB) was inoculated with 5ml of a 0.5 McFarland solution from an overnight culture on a blood agar plate and cultured at 37C for 15h while shaking at 150rpm. After 15h of culturing, bacterial cells were removed by centrifugation (twice at 4000rpm for 10 minutes) and supernatants were collected. The supernatant was filtered with 0.22 m filter (Acrodisc Syringe filter, Poll corporation, USA) to remove the remaining cells/debris and then concentrated by using Ultra-15 100-kDa Amicon centrifugal filter units (Millipore, Billerica, MA, USA) until a final volume of 500l was reached. The filtrate (30 l) was spread onto a blood agar plate to check for any remaining viable bacterial cells.

For further purification of the vesicles, the filtrate was subjected to SEC (Boing etal., 2014). Samples were fractionating into 24 samples of 0.5ml each. The vesicles were collected from fraction 7-11, previously identified to be enriched for MVs and separated from free proteins fractions (Boing etal., 2014; Volgers etal., 2017), pooled and once more concentrated to 500 L by using Ultra-15 100-kDa Amicon centrifugal filter units for 10 minutes at 4000x g. Subsequently vesicles were stored in PBS at -80C until further analysis.

### Vesicles Isolation From Fecal Samples

The steps for optimizing a protocol for isolating heterogeneous bacterial vesicle populations from fecal samples (fMV) were based on previous research performed by Benedikter etal., in which a protocol was designed to isolate MVs from cell culture media (Benedikter etal., 2017). Briefly, 0.5 gram of frozen feces (-80C with less than two weeks storage) was dissolved in 10ml filtered phosphate buffered saline (PBS). Two rounds of centrifugation were applied (15min., 5000rpm, 4C) to remove solid debris. After centrifugation, the debris pellet that contain bacterial cells was resolved in lysis buffer (ASL buffer, QIAamp DNA Stool Kit 51504, QIAGEN, Hilden, Germany) for further bacterial DNA isolation, while the supernatant was filtered by the consecutive use of four filters with different pore sizes: 5.0, 1.2, 0.45 m (Acrodisc syringe filters, Pall Life Sciences, U.S.A) and 0.2 m (Minisart NML syringe filter, Sartorius Stedim Biotech, Germany). For DNA analysis, in those experiments where we determined the bacterial origin of the fMV by PCR, an extra ultracentrifugation step (40,000 rpm, rotor Type 90Ti, 2.5h) was included before ultrafiltration to pellet membrane vesicles and keep free DNA separated in the supernatant. For functional studies vesicles were separated from small molecules by loading the original flow through onto a filter with a molecular weight cut-off of 100 kDa (Amicon Ultra 15ml Centrifugal Filter Unit, Merck Millipore, Billerica, USA) and concentrated it to 250l (45min., 4000 rpm, 4C). The filter membrane was additionally rinsed with 250 l sterile PBS in order to achieve complete fMVs recovery, and an end volume of 500 ul was used for the next step.

The next step involved purification of the concentrate by separating the vesicles from free protein. This was achieved by size exclusion chromatography (SEC) with 10ml CL-2B sepharose columns (GE Healthcare, Eindhoven, the Netherlands). The concentrated supernatant was loaded onto the column and fractions of 0.5ml were immediately collected in Eppendorf tubes. In total, 24 fractions of 0.5ml were collected per sample.

### Vesicles Quantification and Validation

To validate the efficiency of this isolation method of vesicles from fecal samples, both the protein and vesicle content of the obtained SEC fractions was analyzed (we used samples from two individuals for verifications). Protein content was investigated with a Bradford protein assay performed according to manufacturers instructions (Bio-Rad Laboratories). To quantify the absolute vesicle content of each isolate, we used a tunable resistive pulse sensing (TRPS)-based analysis technique (qNano Gold, Izon Science Ltd., Oxford, UK using Izon Control Suite Software v3.2). Measurements were performed with the following settings: an NP150-pore with a stretch of 47mm, a transmembrane voltage of 0.68 V-0.74 V, and a pressure of 20 mbar. The samples were calibrated with 200 nm polystyrene calibration beads that were diluted in Q solution (provide by Izon company). For these analyses, we selected those isolates with clear separation between vesicles and proteins which were fractions 7-11 and further applied for all fecal samples, pooled the fractions obtained by SEC, and stored them at -80C until further analysis.

### Visualizing fMVs by Electron Microscope Cryo-TEM

Three microliters of isolated vesicles were applied to a glow-discharged holey carbon grid before blotting against filter paper to leave only a thin film spanning the grid holes. The sample was kept at 95% humidity before plunge-freezing in liquid ethane using a Vitrobot (FEI, Eindhoven, The Netherlands). The vitreous sample films were transferred to a Tecnai Arctica cryo-Transmission Electron Microscope (ThermoFisher, The Netherlands). The images were taken at 200 kV with a Falcon camera (ThermoFisher, The Netherlands).

### Flow-Cytometric Analysis

Preparation of Antibody-Coated Latex Aldehyde Beads. This method, originally described for the determination of host derived vesicles (Ostrowski etal., 2010), was adapted by our lab to study MVs released by specific bacterial species (e.g. *P. aeroginosa* and *M. catarrhalis*) (Volgers etal., 2017). Overall, 4 different bead sets were prepared as follows: a total of 1x10^8^ 4-M aldehyde-sulfate beads was washed in 150l MES buffer (all washing steps were performed at 3000 x *g* for 10 minutes) and coated with 25 g antibody against outer membrane protein A (OmpA)(abx110631, Abbexa, Cambridge, UK) and lipopolysaccharide (LPS) (ab8467, Abcam, Cambridge, UK) for Gram-negative bacteria, or lipoteichoic acid (LTA) (ab20344, Abcam) for Gram-positive bacteria overnight at 4C while keeping the solution under constant agitation at 6500 rpm. Furthermore, beads were also coated with a combination of CD9/CD63/CD81 for the detection of host-derived vesicles (in a total of 170 l), After coating, the remaining free binding sites on the beads were blocked by washing the beads 3 times with 0.22-M filtered PBS with 4% (w/v) bovine serum albumin (BSA). Then the beads were resuspended and kept in a total of 500 l storage buffer (PBS with 0.1% (v/v) glycine and 0.1% (w/v) sodium azide) at 4C.

Bead-Based Flow-Cytometry. MVs either derived from bacterial culture or fecal samples were conjugated with coated beads and incubated overnight at room temperature under constant agitation at 6500 rpm. After overnight vesicle capture, the beads were washed twice with 0.22-M filtered PBS with 2% (w/v) BSA followed by a final incubation with an analogous secondary PE-conjugated antibody for 1h at room temperature under constant agitation with light protection. Then, the beads were washed twice in 0.22-M filtered PBS with 2% (w/v) BSA, whereafter the pellets were re-suspended in 200 l PBS and analyzed by flow-cytometry on a FACS Canto (BD Bioscience, Franklin Lakes, NJ, USA). Analyses were performed using FACS Diva Software. Single beads were gated on their forward and side scatter. The quartile distribution within a dot plot, based on the fluorescent intensity of single beads, was then used to calculate the relative fluorescent intensity unit. Quartile gate 4 was set on 2% for beads incubated with PBS. The relative fluorescence intensity unit (RFU) was then calculated by multiplying the percentage and the fluorescence intensity of the positive beads in quartile 4.

We additionally determined the specificity of the antibodies by mixing MV from *S. aureus* and *E. coli* in different ratios (*S. aureus/E. coli*: 80:20, 50:50 and 20:80) before incubation with the coated beads and performed the bead-based assay.

### Detection of the Most Common Phyla in the Gut by q-PCR

Origin of feces-derived vesicles was determined by amplification of the bacterial DNA by PCR for most common phyla in the gut using primers and PCR program reactions as previously described (*Firmicutes, Bacteroidetes, Actinobacteria, -Proteobacteria and -Proteobacteria*) (Armougom etal., 2009; Bacchetti De Gregoris etal., 2011). MV-DNA were extracted by heating extraction (at 95 C for 7min) from purified vesicles (isolated by ultrafiltration and SEC in addition to an ultracentrifugation step) to obtain surface and internal associated- vesicles DNA, and directly applied into PCR analysis. The bacterial DNA was isolated from the pellet of the fecal samples by using QIAamp DNA Stool Kit 51504 (QIAGEN, Hilden, Germany) according to the manufacturers instructions and eluted in a final volume of 200 L. The DNA concentration was then measured with Qubit 3 Fluorometer (ThermoFisher Scientific). Two l of target DNA (bacterial and vesicles) was used to perform a real-time 16s qPCR on *Firmicutes, Bacteroidetes, Actinobacteria, -Proteobacteria* and *-Proteobacteria*. Results were regarded as positive if Ct values were below those of the negative controls.

### Immunoreactivity of Feces-Derived Vesicles

#### Cell culture

The human monocytic THP-1 cell line (ATCC-TIB202) was maintained in RPMI1640 (Sigma, St. Louis, MO, USA) supplemented with 10% FCS (Lonza, Verviers, Belgium), glucose (22.5%), sodium pyruvate (100mM), and -mercaptoethanol (25mM) and cultured at 5% CO2 and 37C. For monocyte differentiation, cells were seeded at 1*10^4^ cells/wells in a 96-wells plate and stimulated for 48 hours with 100nM phorbol 12-myristate 13-acetate (PMA; Sigma, St. Louis, MO, USA).

After differentiation, all PMA-containing medium was replaced for 6h with normal culture medium and cells were stimulated for 24h with 10^6^-10^8^ particles/well of feces-derived vesicles. As a negative control, cells were left unstimulated. LPS was used as a positive control for the experiments. After 24h supernatants were collected and centrifuged for 10min at 1200 rpm to remove cells/debris and subsequently stored at -20C for future cytokines analysis.

#### TNF- measurements

As indicator for the immunoreactive potential of the fMVs we measured the release of TNF- with a human TNF- ELISA (Enzyme-linked Immuno Sorbent Assay; human Ready-Set-Go TNF- ELISA kit, eBiosciecnce, 88-7346-86, Affymetrix eBioscience, range 8-500 pg/ml) using manufacturer instructions. All samples including positive and negative control were diluted 1:20. Optical density was measured at 450nm-570nm reading using a Biotek Instruments Powerwave X Microplate Reader.

### Statistical Analyses

All statistical analysis was performed on Graph-Pad Prism 5 Software (Graph-Pad, San Diego, CA, USA). Differences were considered statistically significant when p0.05. An unpaired t-test was performed for the statistical analysis of the variance between the means of 2 groups. Linear regression with 95% confidence error was used for the correlation. All additional tests are stated under their corresponding figures.

## Results

### Vesicles Isolation From Fecal Samples and Bacterial Culture

**Figure 1:**
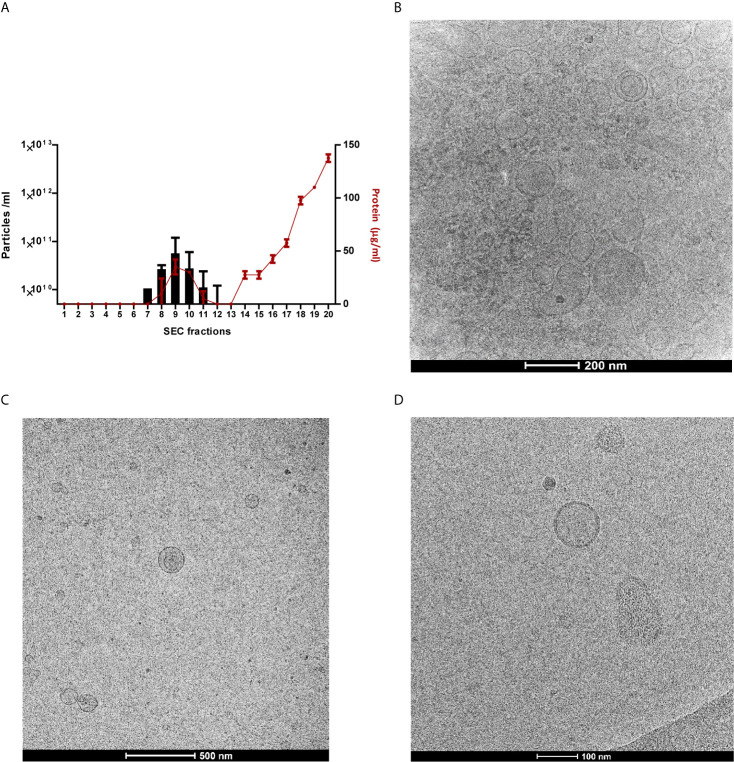
Successful isolation and segregation of particle- and protein-containing fractions. Measurement of particle concentrations and evaluation of vesicle like properties. **(A)** Protein concentrations of all 20 individual SEC fractions (two fecal MV isolates used for verification), determined by Bradford Assay in red dots, overlaid with particle concentrations of fractions 7-11, determined by TRPS in bars, results represent as mean SD. **(B)** TEM images: vesicles shown after ultrafiltration and SEC which reveal the successful isolation procedure of one fecal MVs **(C)** TEM image of the sample when an ultracentrifugation step added, **(D)** TEM image of monoculture-derived vesicles with size ranging from 70-200 nm.

Since the number of studies investigating fMVs is limited we first had to develop a reliable and reproducible protocol for vesicles isolation from feces. This protocol involved a few adaptations of our previously published method for isolation of extracellular vesicles from cell culture medium (Benedikter etal., 2017) and encompassed some additional filtration steps to remove remaining components of fecal material (see M&M section). Finally, after SEC 20 fractions were checked for presence of vesicles and fractions 7-11 were found to have the highest concentration of vesicles (**Figure 1A**). To confirm that these samples predominantly contained vesicles and not proteins, a Bradford assay was performed in combination with tunable resistive pulse sensing (TRPS), see Supplementary **Figures S1A** and **B**. Additionally, with cryo-TEM we also confirmed by visualization the present and size of vesicles. Moreover, we showed that an additional ultracentrifugation step provided even a more pure population of vesicles (**Figures 1BD**). All the samples were standardized to 10^8^ particles/ml, for further use in the following experiments.

### Analysis of Vesicle Origin in Fecal Samples by Bead-Based Flow-Cytometry

**Figure 2:**
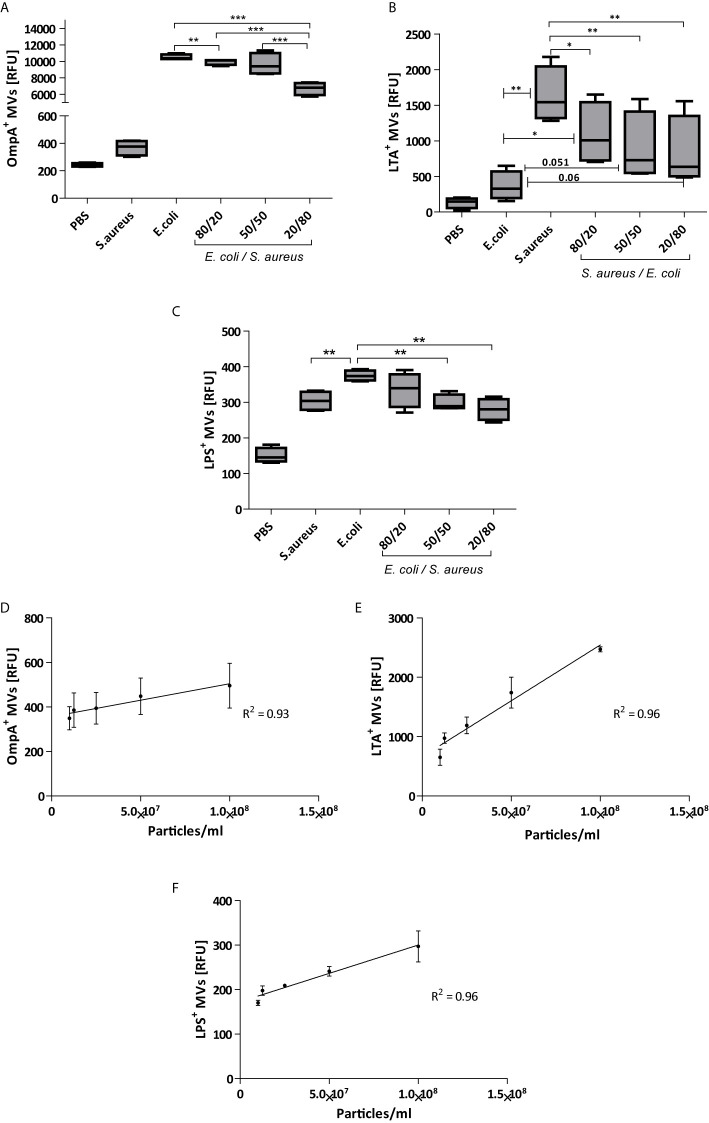
Validation of bead-based flow cytometry on monoculture-derived bacterial vesicles. Vesicles from *E. coli* were clearly captured by beads coated with antibodies against the Gram-negative marker OmpA **(A)** and to a lesser extent by beads coated with anti-LPS antibodies **(C)**. Alternatively, vesicles form *S. aureus* were preferable captured by beads coated with anti-bodies against the Gram-positive marker LTA **(B)**. Specificity was also shown when vesilces form either *E. coli* or *S. aureus* were mixed in different ratios before incubation with different labelled beads. The correlation of bead-based flow relative concentration and absolute numbers of vesicles by TRPS analysis **(D)** OmpA, **(E)** LTA, and **(F)** LPS. Results based on 4 independent experiments. (*p<0.05, **p<0.01, ***p<0.001) Data are represented as Whisker box: Min to Max.

TRPS assay is widely used for vesicle quantification as it provides an unbiased and label-free estimation of the number and size-distribution of the vesicles in an isolate. However, since the complex population of vesicles in fecal samples does not contain only bacterial membrane vesicles but also host-derived vesicles, a more detailed analysis is necessary to determine the ratio between both types of vesicles. As we are predominantly interested in the bacteria-derived membrane vesicles in feces, we optimized the bead-based technique which we have previously developed to measure extracellular vesicles. To determine the (relative) content of Gram-negative and Gram-positive MVs, we coated latex beads with antibodies directed against OmpA or LPS (Gram-negative) or lipoteichoic acid (LTA, Gram-positive). To check the specify of antibodies, we first validated our assays with membrane vesicles isolated from *Escherichia coli* and *Staphylococcus aureus* monocultures. When beads were coated with anti-OmpA, a strong positive signal was obtained with MVs isolated form *E. coli* monocultures, while the signal for MVs form *S. aureus* was significantly less. The reverse result was obtained when beads were coated with anti-LTA antibodies. To validate the specificity of our assay, *E. coli* and *S. aureus* MV were mixed in different ratios (80:20, 50:50, 20:80) and analyzed with beads coated with either anti-LTA of anti-OmpA antibodies. **Figures 2A, B** show a ratio-dependent decrease in the relative fluorescence when *E. coli* MV samples were spiked with *S. aureus* MV and vice versa. These data confirm the specificity of our bead-based assay. It has previously been shown that MV from *S. aureus* contain protein A, which binds to IgG *via* Fc region (Gurung etal., 2011), and this may explain the non-specific binding of the LPS and OmpA antibodies. In both cases (**Figures 2A, C**) the RFU was above the baseline values (*S. aureus* ~350 vs PBS ~200), and this may be the results of the non-specific binding of IgG antibodies to protein A.

We also determined the correlation between the absolute (determined by TPRS) and the relative concentration (bead-based flowcytometry) of MV from either Gram-possitive or Gram-negative MVs (**Figures 2DF**). Linear regression of the slopes of both *E. coli* and *S. aureus* revealed R^2^ values close to 1 (*S. aureus* R^2^ = 0.96 and *E. coli*: R^2^ = 0.93 for OmpA, R^2^ = 0.96 for LPS). This strong correlation demonstrates the applicability of the bead-based flow cytometry assay for semi-quantitative analyses. Furthermore, TRPS-based analysis in combination with bead-based flow cytometry are useful tools to infer the relative concentrations of vesicle subpopulations.

### Bead-Based Flow Cytometry for the Determination of Bacterial and Host-Cell Vesicles Isolated From Fecal Samples

**Figure 3:**
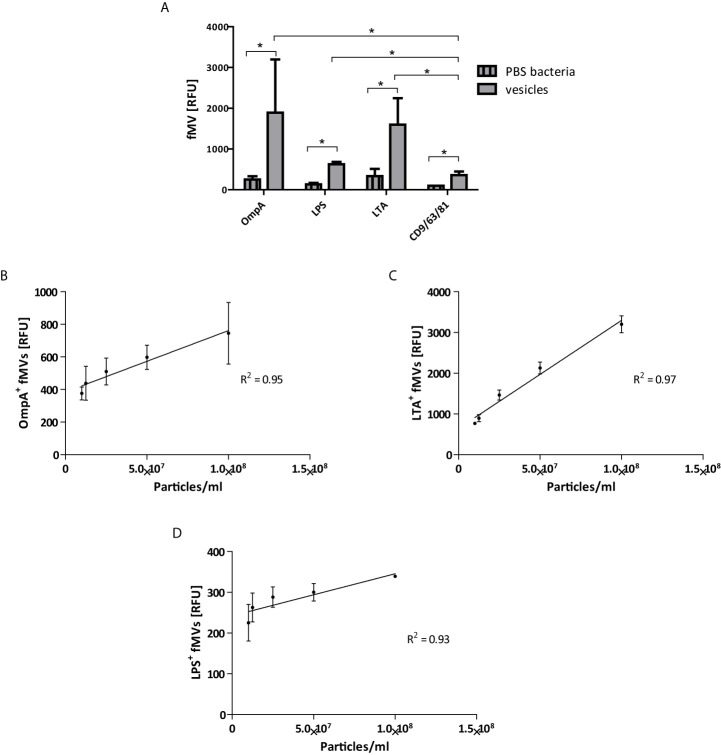
Bead-based flow cytometry analysis on fecal-derived vesicles. **(A)** labeled beads were incubated either with 108 of isolated vesicles from feces, or PBS as a negative control. Vesicles labeled with bacterial markers, (OmpA, LPS and LTA), show high positive signals compared to human exosomes markers (CD9/CD63/CD81) (total of 4 fMVs samples). The correlation between absolute numbers of vesicles determined by TRPS and relative concentration by bead-based flow cytometry analysis of feces-derived vesicles **(B)** OmpA, **(C)** LTA, and **(D)** LPS. (*p<0.05) (data are represented as mean SD n=4).

After validation of the bead-based assay on monoculture vesicles we tested the assay with the aforementioned antibodies on vesicles isolated from fecal samples. Vesicles were isolated by ultrafiltration followed by SEC. **Figure 3A** shows that fMVs from both Gram-negative and Gram-positive bacteria could be detected in fecal samples. Interestingly, when using beads coated with a combination of CD63/CD81/CD9 we could also demonstrate the presence of human extracellular vesicles (hEV) in fecal samples (**Figure 3A**) although the numbers were importantly lower than the fMVs, indicating that the majority of vesicles observed in the isolated samples were bacterial in origin (fMVs: hEVs within individuals, **Figure S2**).

Similar to monoculture MVs, linear regression of slope indicates a strong correlation between the absolute and relative concentration as seen in **Figures 3BD**. Thus, the combination of the bead-based assay with the use of aforementioned markers and a TRPS-based analysis provides a method to clearly segregate mixed populations of feces-derived vesicles.

### Origin of Feces-Derived Vesicles

**Figure 4:**
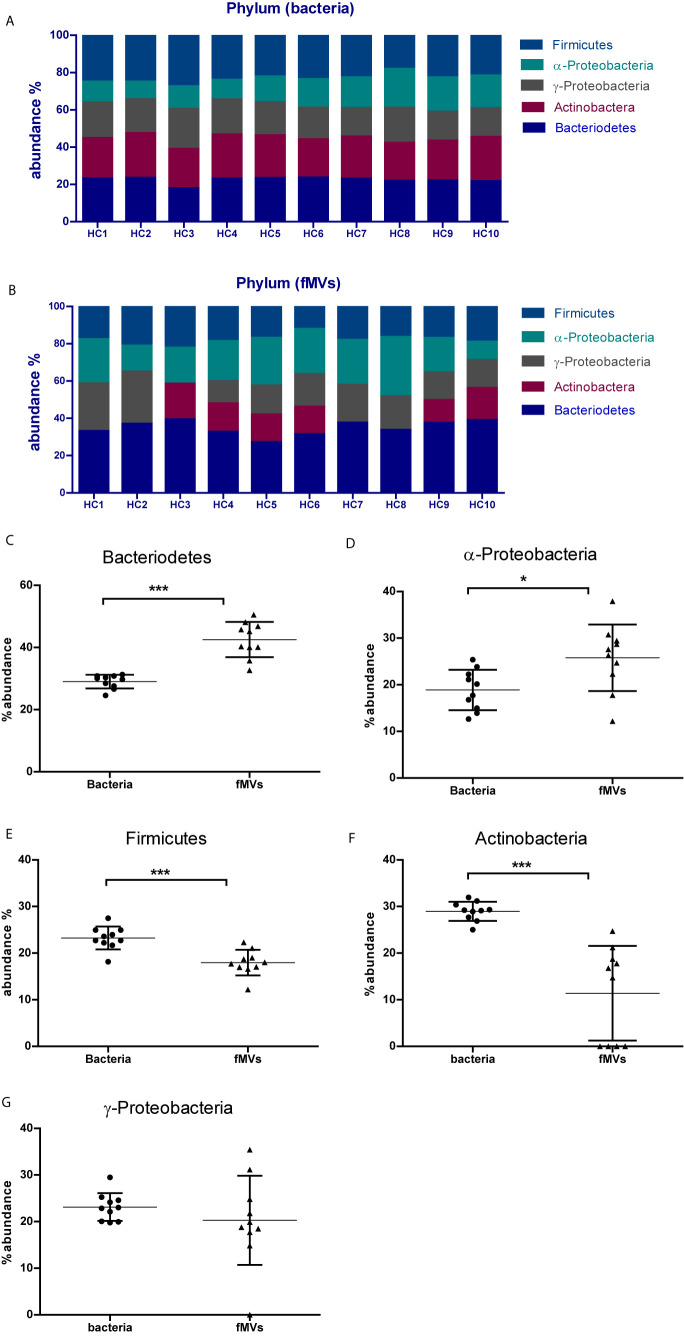
Determination of the bacterial origin of fecal-derived vesicles in 10 samples from healthy volunteers: **(A)** Relative abundance of bacteria of the most common five phyla in all 10 samples, each bar represents one sample. **(B)** Relative abundance of fMVs based on their bacterial origin. Differences in abundance between bacterial cells and fMVs is shown in panels **(CG)**. **(C)** Bacteroidetes, **(D)** -Proteobacteria, **(E)** Firmicutes **(F)** Actinobacteria **(G)** -Proteobacteria. (*p<0.05, ***p<0.001). (Data are represented as mean SD ). Unpaired t test with Welchs correction performed for statistical purpose.

To further verify the origin of the isolated fMVs, a real-time qPCR was performed on DNA isolated form the fMVs, targeting the most abundant commensal phyla *Firmicutes, Bacteroidetes, Actinobacteria, -Proteobacteria* and *-Proteobacteria.* To determine whether the ratios of the fMVs were comparable with the ratios of the parent bacteria in individual fecal samples, real-time q-PCR was also performed on isolated bacterial DNA. Overall, in the bacterial fractions all tested phyla were detected around 20% with slightly higher percentages in case of *Bacteroidetes* and *Firmicutes*, although some differences were detected between individuals (**Figure 4A**). Interestingly, in contrast to bacterial contents, analysis of the vesicle contents revealed different abundance ratios between phyla indicating that the release of vesicles might be species-specific under certain conditions (**Figure 4B**). For example, *Bacteroidetes*-derived vesicles are relatively abundant (~35%) in all individuals. Yet, *Actinobacteria*-derived vesicles could only be detected in 6/10 samples despite the presence of a positive signal in the bacterial DNA fraction. Likewise, in one sample no *-Proteobacteria*-derived vesicles could be detected despite the presence of bacterial DNA. Overall, the relative abundance of *Bacteroidetes*- and *-Proteobacteria*-derived fMVs was significantly higher in fecal samples when compared to the bacterial content, while the opposite was true for *Firmicutes-* and *Actinobacteria*-derived fMVs. No significant differences were noted between the ratios for *-Proteobacteria*-derived fMVs or bacteria.

### Inflammatory Response to fMVs

**Figure 5:**
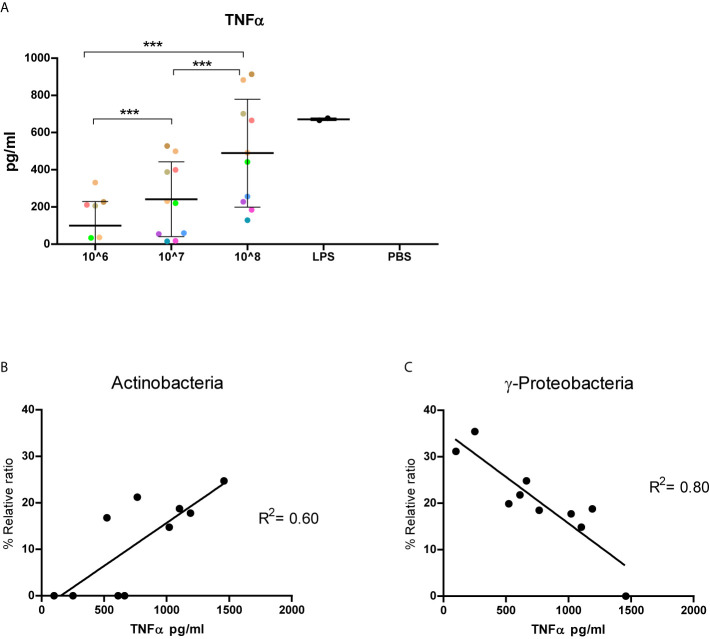
Immunoreactivity of fecal-derived vesicles and implication of bacterial origin. Results of 10 healthy individuals: **(A)** TNF- released by THP-1 cells following exposed to vesicles (10^6^-10^8^/well) isolated from fecal samples from 10 healthy volunteers (each color represent an individual). Interestingly, TNF- response is markedly variable between individuals. **(B, C)** the correlation between phylum composition and corresponding TNF- responses of 10^4^ THP-cells stimulated with 10^8^ particles /well. A positive correlation can be seen in for Actinobacteria (**B**, R^2^ 0.60, p<0.05) while a strong negative correlation was observed for -Proteobacteria (**C**, R^2^0.80, p <0.05). (***p<0.001). Data represent as mean SD.

Next, we assessed immunoreactive characteristics of the isolated fecal MVs by exposing THP-1 macrophages for 24h to 10^6^-10^8^ particles/well of the isolated vesicles from the healthy volunteers. When exposed for 24h to fMVs isolates dose-depend amounts of TNF- were released (**Figure 5A**). The results confirmed the immunoreactive potential of the heterogeneous bacterial fMVs population. Interestingly, fMVs isolates composed of different ratios of bacterial fMVs, which were standardized for vesicle concentration, also showed substantial variation in their corresponding TNF- responses.

### Correlation Between TNF- Production and the Origin of the Vesicles

In order to understand the effect of fMVs origin on TNF- response, a correlation analysis was performed to determine whether the percentage of a specific fMVs type correlated with the amount of TNF- released. A significant positive correlation was found between the percentage of *Actinobacteria*-derived MVs and the amount of TNF- released by THP-1 cells following exposure for 24h (R^2^ = 0.60, p=0.008) (**Figure 5B**). In contrast, fMVs released by *-Proteobacteria* showed a significant negative correlation (R^2^ = 0.80, p=0.0005) **Figure 5C**. Surprisingly, no correlation was found between the percentage of *Bacteroidetes-, Firmicutes- or -Proteobacteria*-derived fMVs in each sample and the amount of TNF-.

## Discussion

Various studies have demonstrated a variety of functions, including immunoreactive potential and inter-bacterial interactions of MVs from selected individual bacterial taxa which all belong to the human commensals (Schertzer and Whiteley, 2013; Kaparakis-Liaskos and Ferrero, 2015; Patten etal., 2017). However, despite extensive knowledge about the gut microbiome at the bacterial level the composition, characteristics and function of the MV population in fecal samples has not been studied before. In this study, we characterized these fMVs and determine to some extent their origin. Initially, based on cryo-TEM images and vesicles quantifications we developed a reproducible isolation protocol for these fMVs. Also, we characterized this heterogeneous population from healthy volunteers by determining the origin of the fMVs in terms of bacterial phyla and their immunoreactive properties.

Feces are a complex combination of undigested carbohydrate, fiber, protein, inorganic components and fat which all comprise the remainder of the daily food intake, but the main component of feces is bacterial biomass (25-50% of dry mass) (Rose etal., 2015). To remove all the unwanted components we performed several centrifugation and (ultra-)filtrations steps to remove debris and small molecules, and finally size exclusion chromatography for further purification of the vesicles. **Figure 1** clearly demonstrates that we were able to isolate a pure population of vesicles. In a recent paper we already demonstrated that a similar technique can be used to isolate extracellular vesicles from cell culture medium and here we confirm the applicability of this technique for fecal samples. Wu and colleagues (Wu etal., 2019) recently published a paper describing a comparable method to isolate vesicles from fecal samples which was solely based on (ultra-)centrifugation (UC). In our hands, however, the yield is significantly higher when using SEC in comparison to UC (Benedikter etal., 2017). Furthermore, UC is not recommended for functional studies since it has been shown that additional material may be spun down during centrifugation (Linares etal., 2015). Additional, by using only UC, no clear distinction can be made between MV and host-derived vesicles. This might in particular be important to consider in proteomic studies since we found a significant number of host-derived MV in our samples. Interestingly, Tulkens etal. recently demonstrated that an additional purification step using Optiprep may be well suited to separate eukaryotic vesicles from bacterial vesicles in plasma (Tulkens etal., 2020). Whether this also applies when using fecal material need to be determined in future studies.

As mentioned, with the label-free TRPS techniques it is not possible to determine the origin of the vesicles (e.g. host vs bacterial). To overcome this problem and discriminate between host and bacterial vesicles we used bead-based flow cytometry. Previously we used this method to determine the release of bacterial vesicles in *in vitro* infection models using specific antibodies directed against the bacteria used in the models (e.g*. Pseudomonas aeruginosa* and *Haemophilus influenza*) (Volgers etal., 2017). However, since the bacterial composition of fecal samples is highly divers, a more general approach was chosen to discriminate solely based on the difference between fMVs derived from either Gram-positive of Gram-negative bacteria. To this end we used general surface markers like OmpA and LPS for fMVs derived from Gram-negative bacteria and LTA for Gram-positive fMVs. **Figure 2**, in case of OmpA and LTA, shows that this approach allows us to discriminate between vesicles of different origin. Nevertheless, it should be kept in mind that the numbers as given in the figures do not represent the true numbers of vesicles present in the samples. These numbers are only relative as the RFU might be affected by efficiency by which the respective Ab binds to the beads as well as relative fluorescence of the Ab. However, when these RFU values of serial dilutions were plotted against the actual numbers of vesicles measured in the samples by TRPS, a strong correlation was observed. This indicates that the relative RFU values are representative of true number of vesicles in each sample and allow us to determine the relative contributions of either Gram-positive or Gram-negative fMVs to the total population. A similar correlation between both techniques has been reported earlier both by us (Volgers etal., 2017) and by Suarez etal., who also concluded that the bead-based flow cytometry method is a reliable method for the semi-quantitative analysis of vesicles populations (Suarez etal., 2017).

In the second part of the study, we were interested in the bacterial origin of vesicles present in feces. Over the last decade, a number of studies have addressed the composition of the gut microbiota and currently there is general consensus that the most common phyla in the gut are *Firmicutes, Bacteroidetes, Actinobaceria* and *Proteobacteria*. Nevertheless, significant variability in the relative contributions of these phyla to the total microbiome has been reported in healthy individuals as well as under pathogenic conditions. However, it remains to be determined whether a similar variability can also be found in the composition of the fMVs. In this study, we examined the presence of fMVs derived from the 5 different phyla by specific PCRs and related this to the relative presence of the bacteria themselves. Regarding the latter, all phyla could be detected in all healthy volunteers, and although some variability was observed, no major difference could be detected between individuals. However, when the fMVs composition was examined, a larger variability was detected. E.g. in only 6/10 samples *Actinobacter*-derived MVs were found, while in one sample no *-Proteobacteria* fMVs could be detected. Overall, we found that the relative contribution to *Bacteroidetes*- and *a-Proteobacteria*-derived fMVs to the total population of vesicles was larger than the contribution of the bacteria themselves. Alternatively, the opposite was the case for the phyla *Firmicutes* and *Actinobacteria*, while the overall contribution for *-Proteobacteria* was not different between fMVs and bacteria. These results indicate that there are significant differences in the number of fMVs produced by the individual phyla. A similar discrepancy was earlier reported by Kang et al., who demonstrated by metagenome analyses that not all bacteria in the large intestine of the mouse produce fMVs, yielding a lower diversity than its source bacteria. Moreover, induction of colitis in this mouse model induced a more drastic change in the composition of the fMVs than the bacterial composition suggesting that the production of fMVs is a dynamic process which highly depends on the specific circumstances (Kang etal., 2013). Nonetheless, interpretations should be careful given the fact that the relationship between MV DNA and bacterial DNA might be disproportional because of the presence of both intra- and extravesicular DNA in MVs (Bitto etal., 2017). A longitudinal follow up of differences in fMVs versus bacterial composition is highly recommended to determine their impact on intestinal homeostasis.

Overall, there is compelling evidence that the majority of bacterial MVs elicit an inflammatory immune response (Macia etal., 2019). This is in accordance with our findings showing a dose-dependent increase in the release of TNF- when THP-1 cells were exposed to increasing concentrations of fMVs. Nonetheless, tolerogenic or even anti-inflammatory effects of specific MVs have also been reported. Especially MVs from *Akkermancia municiphila* have been shown to exert anti-inflammatory effects (Kang etal., 2013). Also MVs derived from various probiotics have been shown to exert anti-inflammatory effects. Since our isolates were a mixture of fMVs derived from the entire microbiome, we were only able to determine to overall effect and not the contribution of fMVs derived from specific bacteria. Yet, when we analyzed a possible association between the relative contributions of different phyla to the overall effect, we noticed a positive correlation between the relative abundance of *Actinobacteria*-derived fMVs and the amount of TNF- released by THP-1 cells. In contrast, a negative correlation was observed for *-Proteobacteria*-derived fMVs, which is in line with a previous study on *E. coli* Nissle 1917 (Fabrega etal., 2017). No correlation was observed for fMVs derived from *-Proteobacteria, Bacteroidetes* or *Firmicutes* in the samples and the amount of TNF- released by THP-1 cells. Taken together, our results suggest that interactions either at the inter- or intraspecies level determine the overall contribution of gut microbiome to the (anti-)inflammatory response. As a result of these interactions, symbiosis or dysbiosis can be achieved, and further implications for health and disease need to be studied in more detail.

In conclusion, the present study shows that ultrafiltration in combination with SEC is a useful method to isolate fecal bacterial membrane vesicles. Additionally, TPRS and bead-based flow cytometry analysis are rapid and accurate methods to determine (semi-)quantitatively the mixed number of bacterial vesicles in fecal samples. Moreover, we identified the origin of fMVs at the phylum level and demonstrated that the relative abundance of fMVs is not identical to the relative abundance of bacteria. The importance of these findings, e.g. with respect to dysbiosis and/or inflammation in intestinal diseases like IBD remains to be determined.

## Data Availability Statement

The original contributions presented in the study are included in the article/Supplementary Material. Further inquiries can be directed to the corresponding author.

## Ethics Statement

The studies involving human participants were reviewed and approved by Medical Ethics Committee of the Maastricht University Medical Center (NL31636.068.10). The patients/participants provided their written informed consent to participate in this study.

## Author Contributions

NK and FS conceived and designed the study. NK and RB conducted the experiments. NK analyzed the results. CL-I performed cryo-TEM imaging. NK and FS wrote the first draft. PS and FS edited the manuscript. All authors contributed to the article and approved the submitted version.

## Conflict of Interest

The authors declare that the research was conducted in the absence of any commercial or financial relationships that could be construed as a potential conflict of interest.

## Supplementary Material

Supplementary Figure 1 Concentrations and size distribution of vesicles obtained from **(A)** bacterial culture (*E.coli)*, **(B)** fecal samples without ultracentrifugation, and **(C)** fecal samples with ultracentrifugation step ( q-NANO images). **(D)** MVs concentrations in pooled SEC samples from different origins. Culture SEC: vesicles isolated by SEC from a *E*. *coli* monoculture. Feces SEC: vesicles isolated from feces with SEC. Feces UC+SEC vesicles isolated from feces by ultracentrifugation and SEC. (*p<0.05). Data represent as mean SD.

Supplementary Figure 2 Bead-based flowcytometry analysis of fMVs. Ratio of bacterial vesicles to human vesicles in fecal samples, OmpA+ identifies Gram-negative bacteria , LTA identifies Gram-positive bacteria and human EV were identified by beads coated with a mixture against of antibodies agaiCD9, CD63, and CD81.
