# Supplementary material for: Characterization of Feces-Derived Bacterial Membrane Vesicles and the Impact of Their Origin on the Inflammatory Response
Source: Front Cell Infect Microbiol. 2021 May 7;11:667987. doi: 10.3389/fcimb.2021.667987 (PMC8139245; doi:10.3389/fcimb.2021.667987)
Supplement: Supplementary Figure 1 — Concentrations and size distribution of vesicles obtained from (A) bacterial culture (E.coli), (B) fecal samples without ultracentrifugation, and (C) fecal samples with ultracentrifugation step ( q-NANO images). (D) MVs concentrations in pooled SEC samples from different origins. Culture SEC: vesicles isolated by SEC from a E. coli monoculture. Feces SEC: vesicles isolated from feces with SEC. Feces UC+SEC vesicles isolated from feces by ultracentrifugation and SEC. (*p<0.05). Data represent as mean SD. [file DataSheet_2.docx]

Supplementary figures:

B

C

A


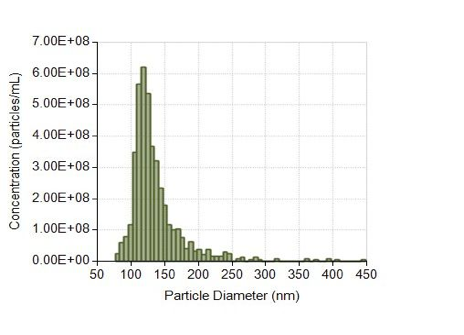

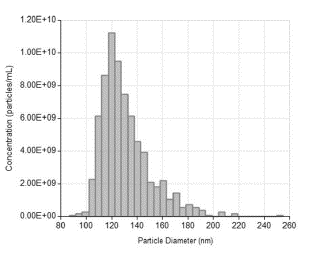

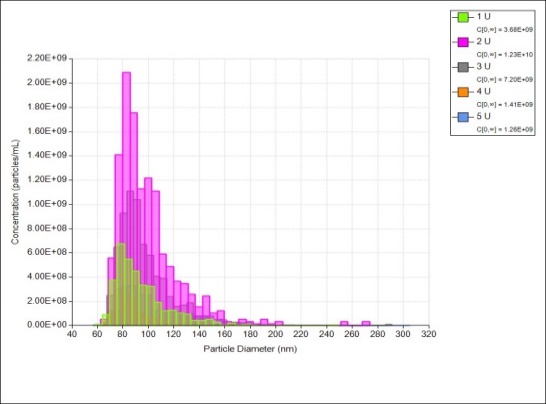

D

**Figure S1**: Concentrations and size distribution of vesicles obtained from (A) bacterial culture (*E.coli)*, (B) fecal samples without ultracentrifugation, and (C) fecal samples with ultracentrifugation step ( q-NANO images). (D) MVs concentrations in pooled SEC samples from different origins. Culture SEC: vesicles isolated by SEC from a *E. coli* monoculture*.* Feces SEC: vesicles isolated from feces with SEC. Feces UC+SEC vesicles isolated from feces by ultracentrifugation and SEC. (* p<0.05). Data represent as mean ± SD.

**Figure S2**: Bead-based flowcytometry analysis of fMVs. Ratio of bacterial vesicles to human vesicles in fecal samples, OmpA+ identifies Gram-negative bacteria , LTA identifies Gram-positive bacteria and human EV were identified by beads coated with a mixture against of antibodies agaiCD9, CD63, and CD81.
